# Fatty Acids in Dry Beans (*Phaseolus vulgaris* L.): A Contribution to Their Analysis and the Characterization of a Diversity Panel

**DOI:** 10.3390/foods13132023

**Published:** 2024-06-26

**Authors:** Roberto Rodríguez Madrera, Ana Campa Negrillo, Juan José Ferreira Fernández

**Affiliations:** 1 Área de Tecnología de los Alimentos, Regional Agrifood Research and Development Service (SERIDA), E-33300 Villaviciosa, Asturias, Spain; 2 Área de Cultivos Hortofrutícolas y Forestales, Regional Agrifood Research and Development Service (SERIDA), E-33300 Villaviciosa, Asturias, Spain; acampa@serida.org (A.C.N.); jjferreira@serida.org (J.J.F.F.)

**Keywords:** common bean, linoleic acid, linolenic acid, palmitic acid, oleic acid, ω-6/ω-3 ratio, heritability

## Abstract

Dry bean (*Phaseolus vulgaris* L.) is a crop of high nutritional interest widespread throughout the world. This research had two objectives. On the one hand, the development and validation of an analytical method to quantify fatty acids in dry beans based on the extraction and derivatization in a single step and later quantification by gas chromatography. On the other, its application to characterize the fatty acid content in a diversity panel consisting of 172 lines. The method was successfully validated in terms of accuracy, precision and robustness. Among the 14 fatty acids that constitute the fatty acid profile of dry bean, the most quantitatively important were linolenic acid, the major fatty acid in all cases, with an average value of 6.7 mg/g, followed by linoleic acid (3.9 mg/g), palmitic acid (2.9 mg/g) and oleic acid (1.5 mg/g). The concentrations of fatty acids in dry bean were influenced by the gene pool, with the Mesoamerican gene pool showing a higher content of palmitic, stearic, linoleic and linolenic acids and the Andean gene pool a higher level of cis-vaccenic acid. Also, the expression of fatty acid content showed high heritability. The information generated constitutes a robust database of interest in food technology, nutrition and breeding programs.

## 1. Introduction

The common bean (*Phaseolus vulgaris* L.) is a legume originally domesticated by pre-Columbian civilizations, in which two main gene pools were identified: the Andean and Mesoamerican. These two gene pools can be distinguished both in the cultivated and wild germplasms [1,2,3]. The high capacity for adaptation of the crop and its nutritional value have led to its widespread cultivation, with beans now being the main legume crop worldwide, with an estimated production of 28.3 million tons in 2022 [4]. Beans are an important source of protein and minerals for specific groups such as vegetarians and low–medium purchasing power populations [5,6]; they are sources of functional compounds such as dietary fiber and antioxidants [7,8,9,10] with beneficial properties for health, such as the prevention of cardiovascular diseases, diabetes and different types of cancer [7,11].

Among the nutritional components of dry beans, their fat content, although low (around 2%), has been identified as beneficial for health due to its contribution of essential fatty acids [5,7]. It has been reported that the main fatty acids (FA) that constitute the triacylglycerides and phospholipids of dried beans are linoleic acid and α-linolenic acid, with a low ratio of linoleic acid to linolenic acid. This low ratio favors reductions in total cholesterol, low-density lipoprotein cholesterol and triglycerides present in the blood lipid profiles of adults, thus helping to reduce the risk of coronary heart disease [12,13,14].

The determination of FA in food matrices is usually carried out in several stages that require prior extraction of the total fat, following different classical procedures. These extraction procedures, such as the Folch method, the Bligh and Dyer method or Soxhlet extraction, are time-consuming, require a high use of solvents (sometimes highly toxic) and extraction with one or another solvent can give rise to different results, both in the extraction performance and the profile of the fatty acids detected [15]. Next, it is necessary to transesterify the FA in the fat into their corresponding fatty acid methyl esters (FAME) or ethyl derivatives, both in a basic medium and in an acidic medium. Finally, the FAMEs are extracted in an organic phase and analyzed with gas chromatography (GC) or high-performance liquid chromatography (HPLC) [16,17,18]. Generally, in this procedure, the results are expressed as a percentage of FA over the total fat content previously estimated by gravimetry, but a direct quantification of the FA of the matrix is not performed.

Alternatively, it is possible to carry out the extraction and derivatization of the FA in different matrices in a single step [15,19,20,21,22]. These one-step methods, which are variants of the Lepage and Roy method [23], simplify the procedure considerably, since they require smaller sample sizes, lower consumption of reagents and time and, in addition, allow the direct quantification of the FA of the sample. Basically, the procedure consists of mixing with the sample in an airtight glass tube the derivatizing reagent (usually BF3/methanol or HCl/methanol) and an organic extractant and heating in a water bath to promote the reaction. Once the reaction is finished, water is added to facilitate the separation of the organic phase, containing the FAME, from the methanolic phase.

Various studies have reported interesting information about the FA in dry beans, pointing out the variability within the species and the influence of growing conditions on its profile [16,18,24,25,26,27,28]. These reports show the FA profiles expressed as a percentage of fatty acid in the total fat previously extracted. However, the fact that these are total fat extractions obtained by different methods can generate differences both in the total fat content and in the relative composition of the FA of the sample, as has already been mentioned. On the other hand, the volume of samples collected is generally small, and the relative information about them is limited to their classification based on their market class, thus encompassing different genotypes that can potentially be generators of different FA profiles. This is why these studies, interesting at a nutritional level, do not allow satisfactory conclusions to be drawn regarding the true diversity of the species or its use in possible breeding programs. For this, it is necessary to have a quantitative characterization of the trait of interest (FA concentration) at the genotype level under controlled conditions, in a sufficiently representative sample of the diversity of the species. This can sometimes be complex to address due to the high workload involved. In this sense, having a validated one-step method would allow the characterization of large collections of samples to be addressed and the establishment of robust databases of interest, both in the field of food technology and in nutrition and breeding programs.

The common bean is a traditional crop in the Iberian Peninsula, and local materials have reported high phenotypic and genotypic diversity. Both gene pools are present in the local germplasm, with the Andean germplasm being the most abundant [29,30]. Also, intermediate material between the two gene pools was described, probably derived from recombination events [31]. In previous studies [10,32,33], our group began the genotypic and the physical–chemical characterization of the Spanish Diversity Panel (SDP), a *P. vulgaris* seed collection that includes most of the phenotypic diversity described in local accessions from Spain, as well as local germplasms of both gene pools described in this species and materials probably derived from recombination between pools.

The aims of this research were to validate a one-step method for FA analysis in dry beans and provide a detailed description of the FA in the species, in order to assess the variations in this group of compounds and provide a robust database to be used in future breeding trials. With this purpose, a sample of 172 lines of the SDP belonging to Andean, Mesoamerican and middle gene pools was analyzed.

## 2. Materials and Methods

### 2.1. Plant Material

One hundred and seventy-two lines of *P. vulgaris*, all of which are included in the Spanish Diversity Panel (SDP) [32], were used in this study. This panel consists of lines established from the local Spanish germplasm, as well as old and elite cultivars. Most of the landraces included are derived from the Spanish common bean core collection [30]. Lines were grown in a greenhouse at the Regional Agrifood Research and Development Service (SERIDA), Villaviciosa, Asturias, Spain in 2018 and in a field trial in 2023 (43°29′01″ N, 5°26′11″ W; elevation 6.5 m). In both cases, a completely randomized design was used with one replicate per line and ten plants distributed in a 1 m row per plot. The dry pods were manually harvested and threshed. The dry seeds were kept under controlled conditions (−20 °C under vacuum) until they were analyzed. Based on 3099 single-nucleotide polymorphism markers (SNPs), the lines were classified according to their genetic pool as Andean, Mesoamerican or middle for those lines coming from crossing between Andean and Mesoamerican parents [32].

Dry beans (50 g) were milled in a coffee grinder, and the powders were sieved through a standard number 18 sieve. The conditions described in Section 2.3 were applied to these flours to determine the fatty acid content. Two extractions were carried out for each sample.

### 2.2. Reagents and Standards

Acetyl chloride, pure standards of palmitic, oleic, cis-vaccenic, linoleic, linoleic, stearic, nonadecanoic fatty acids and the Supelco 37 Component FAME mix were supplied by Merck (Darmstadt, Germany). Methanol and hexane HPLC grade were supplied by Panreac (Barcelona, Spain).

The methylation mixture (methanol/acetyl chloride, 20:1 *v*/*v*) was prepared by carefully adding 10 mL of acetyl chloride to 150 mL of methanol in an ice bath and adjusting to a final volume of 200 mL. Internal standard (IS) solution was prepared by dissolving 400 mg of nonadecanoic acid in 100 mL of dichloromethane. Both solutions were stored at room temperature in amber glass bottles.

### 2.3. Derivatization and Extraction of FAME

Samples (0.15 g of powder) were placed in glass centrifuge tubes with 2 mL of the methylation mixture, 1 mL of hexane and 50 μL of the IS solution. The tubes were heated in a water bath at 100 °C for 60 min, with shaking every 10 min. After cooling, 2 mL of distilled water was added, the tubes were shaken and then centrifuged at 7000× *g* for 10 min. An aliquot of the upper phase (hexane) was used for chromatographic analysis.

### 2.4. Chromatographic Separation, Identification and Quantitation

The separation, identification and quantitation of FAME were carried out in an Agilent 7890A gas chromatograph equipped with a 5975C mass spectrometer and a flame ionization detector (Agilent Technologies, Palo Alto, CA, USA) coupled with a Maestro MPS XL Multi-Purpose Sampler (Gerstel GmbH & Co., Mülheim an der Ruhr, Germany). The injection was performed in split mode (1/20), which improved the symmetry of the major peaks and allowed the quantification of the minor FA. He was used as the carrier gas (1 mL/min).

The optimization of the separation was carried out in a mid-polarity column, with 100% bonded and cross-linked cyanopropyl as the stationary phase, appropriate for the separation of FAME (CP7420 Select FAME 100 m × 250 µm × 0.25 µm, Agilent, Technologies, Palo Alto, CA, USA). The oven temperature program was as follows: 80 °C (1 min) rising to 160 °C (10 min) at a rate of 8 °C /min, rising to 185 °C (8 min) at a rate of 1 °C /min and rising to 240 °C (25 min) at a rate of 20 °C/min.

The identity of FAME was ascertained using data from mass spectrometry, by comparison and combination of their retention times and mass spectra and confirmed with authentic standards.

Quantification was performed with a flame ionization detector (FID), according to an internal standard method. Fatty acid concentrations were calculated by comparing the peak area of each fatty acid methylester in the sample with the peak area of IS as follows:

C_i_ = (A_i_/A_IS_) × C_IS_/W_S_

where C_i_ is the concentration of the fatty acid in the sample, expressed in mg FA/g of sample; Ai is the area of the peak of interest in the chromatogram; A_IS_ is the peak area of the internal standard; C_IS_ is the amount of internal standard in the sample (mg); and W_S_ is the weight of sample (g).

### 2.5. Extraction Optimization

The optimization of the extraction conditions was carried out following a factorial design of 3 factors: transesterification time (4 levels, 15, 30, 60 and 120 min), sample mass (3 levels: 0.050, 0.150 and 0.300 g) and extraction temperature (2 levels: 80 and 100 °C). The rest of the conditions are described in Section 2.3.

### 2.6. Validation Procedure

The parameters checked for method validation were selectivity, examining chromatographic blanks and quality criteria of analyte peaks; and precision, calculated (RSD%) as repeatability and reproducibility. The accuracy of the method was evaluated in three ways: exhaustive extraction (three consecutive extractions of the plant material), a recovery study conducted by spiking with known concentrations of standards at three levels (low, medium and high) and samples from interlaboratory comparisons (Bureau InterProfessionnel d’Etudes Analytiques (BIPEA), Gennevilliers, France).

The linearity of the extraction and linearity of the detector response were determined jointly by the square correlation coefficients of the calibration curves generated by injection of pure standards of FA previously derivatized according to the optimized extraction conditions (Section 2.3). The instrumental limit of detection and instrumental limit of quantification were estimated as 3 × Sa/m and 10 × Sa/m, respectively, from the residuals of the calibration curve for IS at low concentrations, where Sa is the standard deviation of calibration curve intercept values and m is the slope of the calibration curve y = a + mx.

Method limits of detection and quantification (expressed as μg/g) were calculated from the instrumental limits of detection and quantification previously estimated, taking into account the final volume of extracts (1 mL of hexane) and the mass of bean flour used in extraction (0.15 g).

### 2.7. Data Treatment

To detect significant differences between the levels of the factors evaluated in the optimization of the extraction conditions, a three-way ANOVA was applied: transesterification time, extraction temperature and sample mass. To establish significant differences between the dry bean lines analyzed according to the gene pool and the year of cultivation, a two-way ANOVA was used considering the gene pool as a fixed factor and the year of cultivation as a random factor. Duncan’s test was used to establish significant differences between the means in the factors with more than 2 levels. The programs used were SPSS version 15.0 (SPSS Inc., Chicago, IL, USA) and Design Expert 7.0.0 Software (Stat-Ease, Inc., Minneapolis, MN, USA).

The goodness-of-fit for normal distribution was tested using the Kolmogorov–Smirnov test. The broad-sense heritability (H^2^) for FA content was estimated using the repeatability function of the ‘heritability’ package in R version 4.3.2 [34,35]. H^2^ was estimated at the genotypic level according to the following equation:

H^2^ = Vg/(Vg + Ve/r)

where Vg = [MS(G)–MS(E)]/r; Ve = MS(E); r represents the number of replicates per genotype; MS(G) represents the mean sum of squares for genotype; and MS(E) represents the mean sum of squares for residual error obtained from the analysis of variance.

## 3. Results and Discussion

### 3.1. Validation Method

#### 3.1.1. Chromatographic Separation and Identification

**Figure 1:**
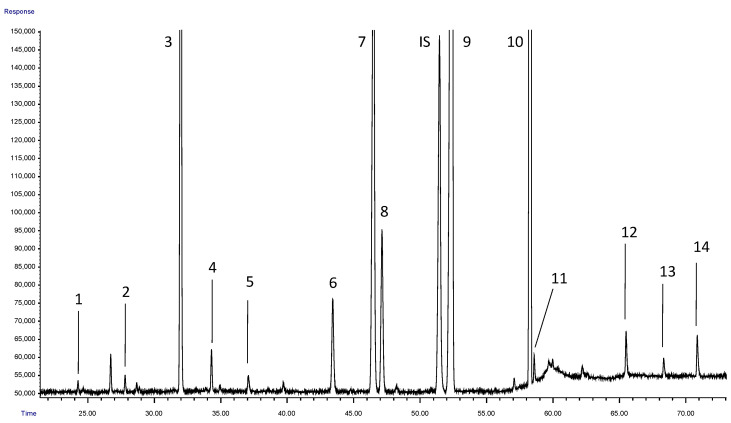
Separation of FAME detected in dry bean extracts. 1: Methyl myristate; 2: Methyl pentadecanoate; 3: Methyl palmitate; 4: Methyl palmitoleate; 5: Methyl margarate; 6: Methyl stearate; 7: Methyl oleate; 8: Methyl cis-vaccenate; 9: Methyl linoleate; 10: Methyl linolenate; 11: Methyl arachidate; 12: Methyl behenate; 13: Methyl tricosanate; 14: Methyl lignocerate; IS: Internal standard (methyl nonadecanoate).

The separation was adequate for all 14 characteristic FAME of dry beans: methyl myristate, methyl pentadecanoate, methyl palmitate, methyl palmitoleate, methyl margarate, methyl stearate, methyl oleate, methyl cis-vaccenate, methyl linoleate, methyl linolenate, methyl arachidate, methyl behenate, methyl tricosanatea and methyl lignocerate. As shown in Figure 1, the method allows detecting differences in the FA content of dry bean, with linoleic and linolenic acids being the most abundant in the species. The identity and purity of the peaks was verified via mass spectrometry and confirmed with the corresponding pure standards. The quality of the spectra of the peaks of interest (≥98%) allowed us to rule out contamination with other FAME or artifacts formed during extraction.

#### 3.1.2. Optimization of Extraction Conditions

The optimization of the extraction conditions was carried out following a factorial design of three factors: transesterification time (15, 30, 60 and 120 min), sample mass (0.050, 0.150 and 0.300 g) and temperature (80 and 100 °C), with two replications per level. In accordance with the literature, HCl/methanol was used as a derivatizing reagent for its low volatility, which maintains shelf-life longevity without special preparation and is low cost; and hexane as extractant with its low toxicity, facilitating the subsequent phase separation [36,37]. The volumes of reagent and extractant were set at 2 mL and 1 mL, respectively, to guarantee the transesterification of the FA and facilitate the collection of the aliquot for subsequent analysis.

The effect of the main factors and the interactions between them was assessed on the six major FAME of *P. vulgaris* (palmitic, stearic, oleic, *cis*-vaccenic, linoleic and linolenic acids) and the total FA content.

As shown in Supplementary Table S1, all the main factors and the second-order interactions were significant (*p* < 0.01), the same behavior being observed for all FAMEs and also for the total sum of FA (TFA). Furthermore, the percentage contribution of each FA to the TFA was similar at the different levels of the experimental design, so the discussion of the results of this section for the variable TFA can be extrapolated to all the major FA of dry bean.

**Figure 2:**
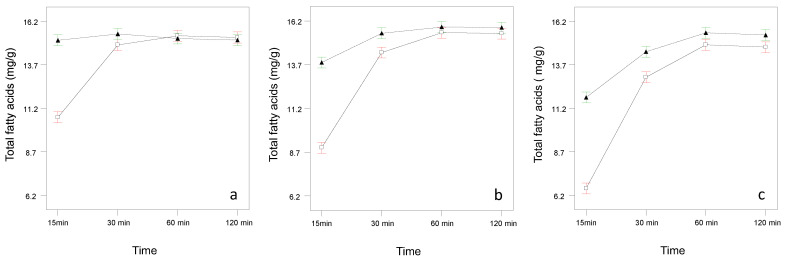
Transesterification time x temperature interaction plots for samples with 0.05 g (**a**), 0.150 g (**b**) and 0.300 g (**c**). White symbols: T = 80 °C. Black color symbols: T = 100 °C.

First of all, it should be noted that the transesterification reaction was significantly favored at a temperature of 100 °C, and this difference is more pronounced for shorter times (significant interaction transesterification time × temperature, Figure 2). Likewise, it was also observed that for masses of 0.05 g the transesterification takes place in 30–60 min, while for masses of 0.3 g at least 60 min was necessary (significant interaction transesterification time × mass, Figure 2).

On the other hand, it should be noted that in the 48 extractions carried out in this section, similar estimations of the IS area were obtained (RSD < 8%), which shows that the esterification of free FA is very fast, even in the less favorable conditions of 15 min at 80 °C. Nevertheless, the results show that since the bean flour is a complex matrix, it requires a greater amount of sample, and longer times are necessary to allow complete transesterification.

Taking these results into account, and given that for a mass of 0.05 g the sensitivity of the method is lower and compromises the quantification of minor FA, it was decided to use a sample quantity of 0.15 g. For this mass, the results show that the minimum agitation should be set at 60 min. Furthermore, it was decided to carry out the transesterification at 100 °C, since at this temperature no degradation in the analytes was detected, and it is not necessary to have a thermostatic water bath.

#### 3.1.3. Linearity and Limits of Detection and Quantification

In order to use the IS response in the quantification of the rest of the FA, it is necessary that the analytical method (extraction and chromatographic analysis) be linear for the different analytes and, furthermore, that the calibration curves do not present significant differences in their slopes. The linearity of the analytical method was evaluated by extraction and analysis of a mixture containing the main fatty acids of interest (palmitic, stearic, oleic, cis-vaccenic, linoleic and linolenic acids) and the IS (nonadecanoic acid), in the usual concentration range, after derivatization under the optimized conditions. For all compounds, the method was linear (R^2^ > 0.999) and the comparison of the slopes using a *t*-test did not show significant differences between IS and the rest of the FA (Supplementary Table S2). Therefore, it is appropriate to use the IS response to quantify the rest of the FA.

The limits of detection and quantification were estimated as 3 × Sa/m and 10 × Sa/m, respectively, from the residuals of the calibration curve for the IS at low concentrations [38], where Sa is the standard deviation of calibration curve intercept values and m is the slope of the calibration curve y = a + mx, and taking into account the final volume of extracts (1 mL hexane) and the mass of dry bean used in extraction (0.15 g sample). The detection and quantification limits were 3 µg/g and 10 µg/g, respectively, suggesting that the proposed method is sufficiently sensitive for the determination of FA compounds in dry beans, in accordance with previous reports [16,25].

#### 3.1.4. Precision, Accuracy and Extract Stability

Precision was calculated in two ways: repeatability and reproducibility, by two operators, on different days and conducting tests independently (three replicates per operator/day). Repeatability (r), estimated for each analyst and compound, was in the range of 0.4–3.2%; reproducibility (R), evaluated as the RSD between analytes, was in all cases ≤5.6% (Supplementary Table S3).

The accuracy of the method was evaluated in three ways: by exhaustive extraction of the plant material, by the standard addition method involving spiking with known concentrations of standards in two matrices and by analyzing six plant material samples from interlaboratory assays.

Exhaustive extraction from two different dry bean matrices showed an average recovery of 90% in the first extraction for the major FA, so a single extraction can be considered satisfactory (Supplementary Figure S1), while mean recovery of 97% was obtained by the standard addition method for the set of analytes and matrices (Table 1).

The analysis of certified reference materials and the participation in intercomparative assays provide greater certainty about the quality of analytical tests. Given the lack of these types of materials for dry bean FA, the suitability of the method was assessed by analyzing other plant matrices from intercomparative tests for which estimated values for total fat content were available. Following the criteria of the FAO [39], the total fat of the samples was estimated from the fatty acids and expressed as triglyceride equivalents according to the conversion tables [40]. Thus, the total fat levels estimated were satisfactory for all matrices, with recoveries of between 92 and 112% and values within the acceptance ranges of the tests (Z score < 2), so the accuracy of the method can be considered satisfactory (Supplementary Table S4).

The stability of the extracts was determined over a period of 15 days, maintaining the extracts at 5 °C. Under these conditions, differences of less than 3% in the analytes were detected, without the appearance of new peaks in the chromatogram as a result of possible degradations or isomerization.

#### 3.1.5. Robustness

Robustness is a measure of an analytical method’s reliability against minor but intentional variations of the method parameters, providing a measure of its reliability during habitual use. Robustness was evaluated using the Youden–Steiner test [41]. In this test, an experiment was used for seven factors: sample mass, heating system, HCl/methanol stability, hexane volume, HCl/methanol volume, IS concentration and chromatographic equipment with eight combinations. Eight chromatographic analyses were carried out to evaluate the effects of those parameters on the major FA and the sum of FAME, following the Youden–Steiner scheme in Supplementary Table S5.

To evaluate the effect of each factor, a difference is determined between the analysis under nominal or routine conditions and the altered conditions (Table 2). This difference (Dif) is the average of the corresponding results (s–z, Supplementary Table S5) for which the factor appears with upper-case letters (nominal values) minus the average of the results in which it appears with lower-case letters (altered conditions) as detailed in Youden and Steiner [41]. If |Dif|>SD×√2, where SD is the standard deviation of the method under conditions of reproducibility, then the factor can affect the veracity of the results, the more so the larger the value of |Dif|.

The test showed a significant decrease in the concentrations of linoleic and linolenic acids, and consequently for the TFA, when a reagent was used one month after its preparation, so this parameter should be considered if it is not prepared frequently (Table 2). Preparing the reagent at least weekly is recommended if it is stored at 20–25 °C in topaz-colored containers. The rest of the factors did not affect the results, so the method can be considered acceptably robust.

### 3.2. Variation of Fatty Acid Content in Bean Seed

The validated method was used to quantify the FA content in *P. vulgaris* seeds. For this, dry beans from 172 lines included in the Spanish Diversity Panel harvested in two contrasting environments (greenhouse and field) were analyzed. This panel brings together wide phenotypic and genotypic variations [32]. All lines showed a similar profile from a qualitative point of view, with 14 FA of which 9 were saturated fatty acids (SFA: myristic, pentadecanoic, palmitic, margaric, stearic, arachidic, behenic, tricosanoic and lignoceric acids) and 5 unsaturated fatty acids (UFA: palmitoleic, oleic, cis-vaccenic, linoleic and linolenic acids), with values that ranged from the limits of quantification of 0.01 mg/g for myristic and pentadecanoic acids, and maximum concentrations of 9.22 and 9.26 mg/g for linoleic and linolenic acids, respectively (Supplementary Table S6).

**Figure 3:**
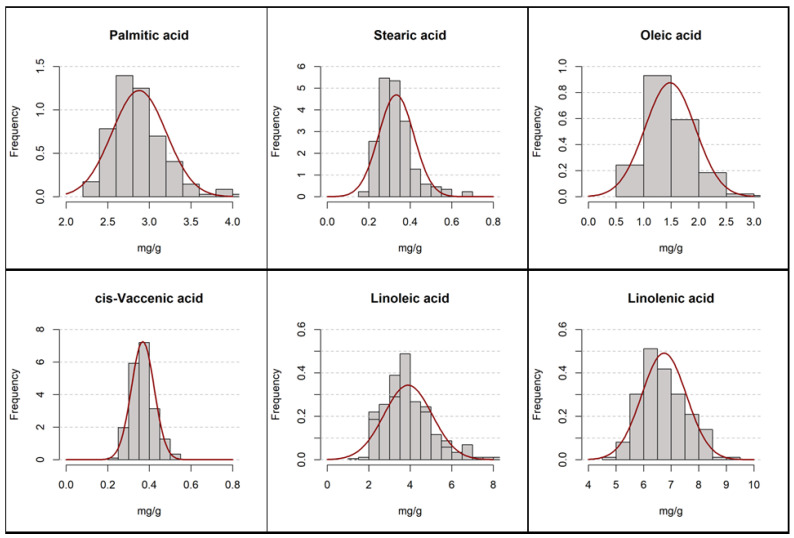
Concentration distributions of the main fatty acids in dry beans.

However, it should be noted that only six of them can be considered relevant from a quantitative point of view, with contributions to the TFA of above 2%: palmitic, stearic, oleic, cis-vaccenic, linoleic and linolenic acids, which together accounted for between 95.6 and 97.8% of the measured total for the individuals in the database. Figure 3 shows the distribution of the content of those six main FA in the bean seed. The results of the normality tests (Kolmogorov–Smirnov) indicate a fit to a normal distribution in all cases. The rest of the quantified FA in no case represented 1% of the total, so their contribution to the total fat content can be considered to be of little relevance for the characterization of the species.

The content of TFA showed important variability within the panel, with values that ranged between 11.22 mg/g (line SDP192, market class Cranberry, year 2023) and 25.76 (SDP004, market class Laran, year 2023) and an average content for the set of samples of 16.20 mg FA/g. These results agree with those reported for total fat values in the species by other authors, with values of 1.3% and 2.5% by weight [5,18,24].

Within the dry bean FA set, the main group consisted of unsaturated fatty acids (UFA), which accounted for 72–80% of the total, which is especially relevant because of the major health benefits of this class of FA [12,42], compared to saturated fatty acids (SFA), which account for between 20% and 28% of the total.

The UFA were mostly polyunsaturated fatty acids (PUFA), with a range of 54–72%, compared to 5–23% for monounsaturated fatty acids (MUFA). These ranges are within the percentages described by other authors in dry beans of different commercial classes [18,24,25,26], and the fact that it is a PUFA-rich food gives it an extra nutritional dimension [5].

The PUFA of *P. vulgaris* are basically constituted by linoleic acid (C18:2 ω-6) and α-linolenic acid (C18:3 ω-3), both essential FAs that must be supplied by food, and low intakes are said to contribute to dermatitis, renal hypertension, arthritis and type 2 diabetes, among other diseases. Moreover, a moderate linoleic acid intake could reduce blood total cholesterol and low-density lipoprotein (LDL) cholesterol, and linolenic acid has been demonstrated to have anti-inflammatory, neuroprotective and antidepressant effects [14,43]. In accordance with other authors [18,24,25,26], these acids were also the most prevalent in the species, linoleic acid accounting for 24% of the total FA and linolenic acid for 42%, with concentrations for linoleic acid ranging from 1.78 mg/g (SDP063, year 2023; market class Dark garbanzo) to 9.22 mg/g (SDP004, year 2023; market class Laran) and for linolenic acid between 4.33 mg/g (SDP042, year 2018) and 9.26 mg/g (SDP031, year 2023; market class Small white). Moreover, the low ω-6/ω-3 ratio detected in the species, ranging between 0.23 and 1.37, may contribute to reducing this ratio in the diet, a measure that could avoid the pathogenesis of many modern diet-related chronic diseases associated with overconsumption of ω-6 PUFAs with a concomitant low intake of ω-3 PUFAs [14,44].

Concerning MUFA, two major acids were detected, oleic acid (C18:1 ω-9) and cis-vaccenic (C18:1 ω-7), representing 9% and 2% of the FA of the species, respectively, in agreement with the reported profiles for different commercial classes of dry bean [18,24,25,26]. Quantitatively, oleic acid content ranged between 0.45 mg/g (SDP166, year 2023; market class Fabada) and 3.95 mg/g (SDP201, year 2023; market class Black opaque), and cis-vaccenic acid between 0.21 mg/g (SDP107, year 2018; market class Brown mottled) and 0.55 mg/g (SDP009, year 2023). Another MUFA detected was palmitoleic acid (C16:1 ω-7), but its contribution to the species profile was not relevant in all cases (mean value = 0.27%). Although oleic acid is associated with human health benefits (limiting the formation of proatherogenic oxidized LDL, having a small blood pressure-lowering effect, possibly improving glucose control and insulin sensitivity) these effects could be related not so much to the action of oleic acid as to the replacement of part of the saturated fatty acid intake with this monounsaturated acid [42]. In any case, the availability of varieties with a higher MUFA content is desirable. On the other hand, the high variability in oleic and linoleic acid content could be of interest for consideration in breeding programs, since different studies in other plant species have shown that increasing the level of polyunsaturated fatty acid can improve seed performance in the field at low temperatures, and seeds with higher oleic content could be more adapted to high temperatures [45,46].

Of the nine SFA quantified (miristic acid, pentadecanoic acid, palmitic acid, margaric acid, stearic acid, arachidic acid, behenic acid, tricosanic acid and lignoceric acid), only palmitic and stearic acids were quantitatively relevant in the profile of the species (Supplementary Table S6). Palmitic acid was the main SFA, representing an average content of 18% of the FA, with concentrations ranging from 2.09 mg/g (SDP192, year 2023, market class Cranberry) to 4.50 mg/g (SDP004, market class Laran), while stearic acid represented a mean content of 2%, with concentrations ranging from 0.18 mg/g (SDP300, year 2023) to 0.75 mg/g (SDP004, year 2023, market class Laran). Nutritionally, palmitic acid can raise total and LDL cholesterol concentrations and could increase coagulation, inflammation and insulin resistance [42], so although the levels of palmitic acid in dry bean are not high, a correct selection of varieties can contribute to a lower intake of this FA.

Qualitatively, the FA profile described for *P. vulgaris* seed is similar to those described for other non-oil legume species consumed throughout the world such as chickpeas, peas, lentils and other minor legume species [17,27,47,48,49,50,51,52,53]. These profiles all contain a small number of FA that are characteristic of such legumes, usually palmitic acid, oleic acid, linoleic acid and linolenic acid, and other less abundant FA, which together allow discriminating differences between species to be established. Compared to other legumes, the FA profile of *P. vulgaris* seed is notable for being mostly linolenic acid, which makes it one of the legumes with the lowest ω-6/ω-3 ratio.

### 3.3. Gene Pool and Fatty Acid Content in Dry Bean

Common bean (*P. vulgaris* L.) comprises two gene pools, Mesoamerican and Andean, which differ in their structures and levels of genetic diversity, both in wild and domesticated populations [54]. The results show significant differences between the gene pool groups (*p* < 0.05) in the content of five of the six major FA (Table 3). On the one hand, the Mesoamerican gene pool was the group with the highest levels of palmitic, stearic, linoleic and linolenic acids. On the other hand, the Andean gene pool group had the highest contents of cis-vaccenic acid and linolenic acid, while the group of middle gene pool samples showed no differences with respect to the Andean group for four of the six major FA. As a consequence of these differences, the Mesoamerican gene pool had the highest levels of TFA, SFA and UFA, and the Andean and middle gene pools had the lowest ω-6/ω-3 ratio.

In addition, significant differences (*p* < 0.05) were detected between years of cultivation for stearic, oleic and linolenic acids (Table 3), which shows the influence of environmental conditions on the FA profile, in agreement with previous reports [28,55].

However, the gene pool × year of cultivation interaction was not significant for any FA or TFA, and the estimated broad-sense heritability (H^2^) values of those six FA were high, ranging from 0.74 (palmitic acid) to 0.87 (linoleic acid) (Table 3), indicating the high genetic component in the expression of the character. In this regard, it is important to note that the classification of the lines as high and low TFA producers remained reasonably stable over the two years of the study, considering the large number of lines managed in this trial (Supplementary Table S6). Thus, 60% of the samples located in the first quartile and 50% of those located in the fourth quartile were maintained in both years.

## 4. Conclusions

A method for accurately quantifying the fatty acids in dry beans (*P. vulgaris*) based on the extraction and derivatization in a single step and quantification by GC-FID has been successfully developed and validated. The analytical characteristics of the method (repeatability, reproducibility, accuracy, linearity, sensitivity and robustness) allow the fatty acids of a diversity panel to be reliably quantified. The analysis of dry beans from 172 lines, collected over 2 years of cultivation, showed wide diversity in the content of fatty acids in the species, with a total fatty acid content of between 11.22 mg/g and 25.76 mg/g, highlighting the high level of polyunsaturated fatty acids that constitute an average of 65% of the fatty acids. Among the 14 fatty acids that constitute the fatty acid profile of dry bean, the most important ones quantitatively were linolenic acid, the major fatty acid in all cases, with a mean value of 6.7 mg/g, followed by linoleic acid (3.9 mg/g), palmitic acid (2.9 mg/g) and oleic acid (1.5 mg/g). The expression of fatty acid content showed high heritability, and the concentration of fatty acids in dry beans was influenced by the gene pool, with the Mesoamerican gene pool showing a higher content of palmitic, stearic, linoleic and linolenic acids and the Andean gene pool a higher level of *cis*-vaccenic acid. The information generated constitutes a robust database that contributes to the characterization of the species, allowing the availability of *P. vulgaris* lines with greater nutritional interest both for direct consumption and in the formulation of food products. Likewise, the information obtained will allow for a more adequate selection of the working lines in future breeding programs and can be used to investigate the genomic regions associated with the synthesis of fatty acids in the species.

## Figures and Tables

**Table 1:** Results of recovery study using standard addition method in dry beans Cornell49292 and A4804.

	Palmitic Acid	Stearic Acid	Oleic Acid	*cis*-Vaccenic Acid	Linoleic Acid	Linolenic Acid
***Line Cornell49242***						
Sample without spiking (mg/g)	3.79	0.46	2.11	0.33	8.22	6.09
Spiking level 1 (mg/g)	1.00	0.17	0.70	0.20	3.10	1.72
Conc ± RSD (n = 3)	4.75 ± 1.28	0.62 ± 1.5	2.78 ± 1.03	0.53 ± 1.94	11.04 ± 1.45	7.87 ± 0.84
*Recovery level 1 (%)*	*96*	*94*	*96*	*100*	*91*	*103*
Spiking level 2 (mg/g)	2.00	0.35	1.39	0.40	6.20	3.40
Conc ± RSD (n = 3)	5.71 ± 0.39	0.79 ± 0.38	3.39 ± 1.26	0.72 ± 1.90	13.97 ± 1.14	9.56 ± 1.41
*Recovery level 2 (%)*	*96*	*94*	*92*	*97*	*93*	*102*
Spiking level 3 (mg/g)	4.00	0.70	2.78	0.80	12.40	6.80
Conc ± RSD (n = 3)	7.59 ± 0.40	1.11 ± 0.66	4.73 ± 1.00	1.11 ± 0.32	19.8 ± 0.36	13.34 ± 0.70
*Recovery level 3(%)*	*95*	*93*	*94*	*98*	*93*	*107*
***Line A4804***						
Sample without spiking(mg/g)	2.78	0.31	1.52	0.37	3.38	7.52
Spiking level 1 (mg/g)	1.00	0.17	0.70	0.20	3.10	1.72
Conc ± RSD (n = 3)	3.74 ± 1.20	0.48 ± 2.03	2.17 ± 0.89	0.56 ± 0.84	6.33 ± 1.46	9.2 ± 0.51
*Recovery level 1 (%)*	*96*	*103*	*93*	*94*	*95*	*98*
Spiking level 2 (mg/g)	2.00	0.35	1.39	0.40	6.20	3.40
Conc ± RSD (n = 3)	4.68 ± 1.20	0.64 ± 2.03	2.84 ± 0.89	0.75 ± 0.84	9.08 ± 1.46	11.01 ± 0.51
*Recovery level 2 (%)*	*95*	*96*	*95*	*95*	*92*	*101*
Spiking level 3 (mg/g)	4.00	0.70	2.78	0.80	12.40	6.80
Conc ± RSD (n = 3)	6.59 ± 0.93	0.98 ± 0.51	4.27 ± 0.11	1.16 ± 0.08	14.85 ± 0.60	14.86 ± 0.49
*Recovery level 3(%)*	*95*	*96*	*99*	*99*	*93*	*108*

**Table 2:** Calculated differences (|Dif|) for fatty acids under the factors established to evaluate robustness following the Youden–Steiner test.

Factor	Palmitic Acid	Stearic Acid	Oleic Acid	*cis*-Vaccenic Acid	Linoleic Acid	Linolenic Acid	Total Fatty Acids
Sample mass	0.099	0.024	0.065	0.010	0.368	0.271	0.876
Heating system	0.054	0.007	0.074	0.014	0.347	0.295	0.821
HCl/Methanol stability	0.134	0.023	0.100	0.014	0.502 *	0.402 *	1.182 *
Volume hexane	0.059	0.016	0.053	0.014	0.341	0.280	0.785
Vol HCl/Methanol	0.081	0.010	0.080	0.013	0.426	0.287	0.894
Internal standard concentration	0.052	0.013	0.033	0.006	0.172	0.169	0.432
Chromatographic equipment	0.025	0.016	0.037	0.002	0.205	0.174	0.391
SD	0.157	0.026	0.086	0.016	0.324	0.235	0.782
SD×√2	0.221	0.036	0.122	0.022	0.458	0.332	1.106

*: Significant difference.

**Table 3:** Contents of fatty acids in dry bean (*Phaseolus vulgaris* L.) according to gene pool and year of cultivation (expressed in mg/g) and broad-sense heritability.

		Palmitic Acid	Stearic Acid	Oleic Acid	*cis*-Vaccenic Acid	Linoleic Acid	Linolenic Acid	TFA	SFA	UFA	MUFA	PUFA	ω-6/ω-3
Gene pool													
*Andean*	*mean*	*2.77 ^a^*	*0.29 ^a^*	*1.43*	*0.38 ^b^*	*3.53 ^a^*	*6.81 ^b^*	*15.69 ^a^*	*3.49 ^a^*	*12.2 ^a^*	*1.86*	*10.34 ^a^*	*0.52 ^a^*
(n = 65)	max	3.38	0.56	3.80	0.51	5.54	8.76	19.72	4.50	15.68	4.18	13.73	0.89
	min	2.09	0.18	0.52	0.28	1.78	5.09	11.22	2.62	8.60	0.85	7.54	0.23
*Mesoamerican*	*mean*	*3.09 ^b^*	*0.37 ^c^*	*1.56*	*0.33 ^a^*	*4.57 ^b^*	*6.83 ^b^*	*17.28 ^b^*	*3.95 ^c^*	*13.33 ^b^*	*1.93*	*11.4 ^b^*	*0.7 ^b^*
(n = 51)	max	4.35	0.71	3.95	0.47	8.30	9.26	25.46	5.61	19.85	4.38	15.47	1.37
	min	2.45	0.22	0.66	0.21	1.97	4.33	13.63	3.10	10.12	1.00	8.37	0.25
*Middle*	*mean*	*2.81 ^a^*	*0.35 ^b^*	*1.47*	*0.39 ^b^*	*3.71 ^a^*	*6.58 ^a^*	*15.81 ^a^*	*3.62 ^b^*	*12.19 ^a^*	*1.90*	*10.28 ^a^*	*0.57 ^a^*
(n = 56)	max	4.50	0.75	2.79	0.55	9.22	8.84	25.76	6.14	19.61	3.18	17.38	1.13
	min	2.24	0.19	0.45	0.25	1.82	4.76	12.37	2.92	9.20	0.77	7.48	0.29
Year of cultivation													
*2018*	*mean*	*2.86*	*0.36 ^b^*	*1.58 ^b^*	*0.38*	*3.80*	*6.62 ^a^*	*16.11*	*3.69*	*12.42*	*2.00 ^b^*	*10.42*	*0.59*
(n = 172)	max	3.89	0.71	3.80	0.53	7.57	8.95	22.17	5.25	16.93	4.18	15.33	1.37
	min	2.24	0.19	0.49	0.21	1.86	4.33	12.37	2.91	9.20	0.79	7.48	0.23
2023	*mean*	*2.89*	*0.31 ^a^*	*1.38 ^a^*	*0.36*	*3.99*	*6.87 ^b^*	*16.29*	*3.65*	*12.64*	*1.79 ^a^*	*10.86*	*0.59*
(n = 172)	max	4.50	0.75	3.95	0.55	9.22	9.26	25.76	6.14	19.85	4.38	17.38	1.37
	min	2.09	0.18	0.45	0.24	1.78	4.76	11.22	2.62	8.60	0.77	7.54	0.25
Gene pool × Year of cultivation		n.s.	n.s.	n.s.	n.s.	n.s.	n.s.	n.s.	n.s.	n.s.	n.s.	n.s.	n.s.
Heritability, *H^2^*		*0.74*	*0.79*	*0.80*	*0.86*	*0.87*	*0.82*	*0.76*	*0.78*	*0.75*	*0.78*	*0.75*	0.93

TFA: Total fatty acids; SFA: saturated fatty acids; UFA: unsaturated fatty acids; MUFA: monounsaturated fatty acids; PUFA: polyunsaturated fatty acids. Different letters mean significant differences at *p* < 0.05. n.s.: not significant. n: number of lines.

## Data Availability

The original contributions presented in the study are included in the article and Supplementary Materials, further inquiries can be directed to the corresponding author.

## Supplementary Materials

The following supporting information can be downloaded at: https://www.mdpi.com/article/10.3390/foods13132023/s1. Supplementary Table S1. Major fatty acids and total fatty acids at the levels of the factorial design. Data expressed in mg/g and in brackets expressed as %. Supplementary Table S2. Analytical characteristics of the calibration graphs of major fatty acids in dry beans. Supplementary Table S3. Repeatability and reproducibility of the method under the optimized conditions. Supplementary Table S4. Method accuracy in samples from interlaboratory assays. Supplementary Table S5. Youden–Steiner test scheme and results of the analysis of the major fatty acids under these conditions. Supplementary Table S6. List of lines included in the Spanish Diversity Panels analyzed in this study and fatty acid composition. Supplementary Figure S1. Concentration (mg/g) and recovery (%) of exhaustive extraction of palmitic acid (C16:0), stearic acid (C18:0), oleic acid (C18:1 ω-9), cis-vaccenic acid (C18:1 ω-7), linoleic acid (C18:2 ω-6) and linolenic acid (C18:3 ω-3) in dry beans of lines Cornell49242 and A4804.

## References

[1] Bitocchi E., Bellucci E., Giardini A., Rau D., Rodriguez M., Biagetti E., Santilocchi R., Spagnoletti Zeuli P., Gioia T., Logozzo G. (2013). Molecular Analysis of the Parallel Domestication of the Common Bean (*Phaseolus vulgaris*) in Mesoamerica and the Andes. New Phytol..

[2] Gepts P., Osborn T.C., Rashka K., Bliss F.A. (1986). Phaseolin-protein Variability in Wild Forms and Landraces of the Common Bean (*Phaseolus vulgaris*): Evidence for Multiple Centers of Domestication. Econ. Bot..

[3] Kwak M., Gepts P. (2009). Structure of Genetic Diversity in the Two Major Gene Pools of Common Bean (*Phaseolus vulgaris* L., Fabaceae). Theor. Appl. Genet..

[4] (2024). FAOSTAT. http://www.fao.org/faostat/en/#data/QC.

[5] Yoshida H., Tomiyama Y., Kita S., Mizushina Y. (2005). Lipid Classes, Fatty Acid Composition and Triacylglycerol Molecular Species of Kidney Beans (*Phaseolus vulgaris* L.). Eur. J. Lipid Sci. Technol..

[6] Diaz S., Polania J., Ariza-Suarez D., Cajiao C., Grajales M., Raatz B., Beebe S.E. (2022). Genetic Correlation Between Fe and Zn Biofortification and Yield Components in a Common Bean (*Phaseolus vulgaris* L.). Front. Plant Sci..

[7] Hayat I., Ahmad A., Masud T., Ahmed A., Bashir S. (2014). Nutritional and Health Perspectives of Beans (*Phaseolus vulgaris* L.): An Overview. Crit. Rev. Food Sci. Nutr..

[8] Messina V. (2014). Nutritional and Health Benefits of Dried Beans. Am. J. Clin. Nutr..

[9] Murube E., Beleggia R., Pacetti D., Nartea A., Frascarelli G., Lanzavecchia G., Bellucci E., Nanni L., Gioia T., Marciello U. (2021). Characterization of Nutritional Quality Traits of a Common Bean Germplasm Collection. Foods.

[10] Rodríguez Madrera R., Campa Negrillo A., Suárez Valles B., Ferreira Fernández J.J. (2020). Characterization of Extractable Phenolic Profile of Common Bean Seeds (*Phaseolus vulgaris* L.) in a Spanish Diversity Panel. Food Res. Int..

[11] Rodríguez L., Mendez D., Montecino H., Carrasco B., Arevalo B., Palomo I., Fuentes E. (2022). Role of *Phaseolus vulgaris* L. in the Prevention of Cardiovascular Diseases—Cardioprotective Potential of Bioactive Compounds. Plants.

[12] Djuricic I., Calder P.C. (2021). Beneficial Outcomes of Omega-6 and Omega-3 Polyunsaturated Fatty Acids on Human Health: An Update for 2021. Nutrients.

[13] Rajaram S. (2014). Health Benefits of Plant-Derived α-Linolenic Acid. Am. J. Clin. Nutr..

[14] Wang Q., Zhang H., Jin Q., Wang X. (2023). Effects of Dietary Plant-Derived Low-Ratio Linoleic Acid/Alpha-Linolenic Acid on Blood Lipid Profiles: A Systematic Review and Meta-Analysis. Foods.

[15] Ruiz-Rodriguez A., Reglero G., Ibañez E. (2010). Recent Trends in the Advanced Analysis of Bioactive Fatty Acids. J. Pharm. Biomed. Anal..

[16] Byrdwell W.C., Goldschmidt R.J. (2022). Fatty Acids of Ten Commonly Consumed Pulses. Molecules.

[17] Ologhobo A. (1983). Varietal Differences in the Fatty Acid Composition of Oils from Cowpea (*Vigna unguiculata*) and Limabean (*Phaseolus lunatus*). Food Chem..

[18] Lo Turco V., Potortì A.G., Rando R., Ravenda P., Dugo G., Di Bella G. (2016). Functional Properties and Fatty Acids Profile of Different Beans Varieties. Nat. Product. Res..

[19] Abdulkadir S., Tsuchiya M. (2008). One-Step Method for Quantitative and Qualitative Analysis of Fatty Acids in Marine Animal Samples. J. Exp. Mar. Biol. Ecol..

[20] Harmanescu M. (2012). Comparative Researches on Two Direct Transmethylation without Prior Extraction Methods for Fatty Acids Analysis in Vegetal Matrix with Low Fat Content. Chem. Cent. J..

[21] Xiao S., Li H., Xu M., Huang K., Luo Z., Xiao L. (2021). A High-Throughput Method for Profiling Fatty Acids in Plant Seeds Based on One-Step Acid-Catalyzed Methylation Followed by Gas Chromatography-Mass Spectrometry. Biotechnol. Biotechnol. Equip..

[22] La Cruz García C., López Hernández J., Simal Lozano J. (2000). Gas chromatographic determination of the fatty-acid content of heat-treated green beans Cultivars. J. Chromatogr. A.

[23] Lepage G., Roy C.C. (1984). Improved recovery of fatty acid through direct transesterification without prior extraction or purification. J. Lipid Res..

[24] Sutivisedsak N., Moser B.R., Sharma B.K., Evangelista R.L., Cheng H.N., Lesch W.C., Tangsrud R.R., Biswas A. (2011). Physical Properties and Fatty Acid Profiles of Oils from Black, Kidney, Great Northern, and Pinto Beans. J. Am. Oil Chem. Soc..

[25] Caprioli G., Giusti F., Ballini R., Sagratini G., Vila-Donat P., Vittori S., Fiorini D. (2016). Lipid Nutritional Value of Legumes: Evaluation of Different Extraction Methods and Determination of Fatty Acid Composition. Food Chem..

[26] Khrisanapant P., Kebede B., Leong S.Y., Oey I. (2019). A Comprehensive Characterisation of Volatile and Fatty Acid Profiles of Legume Seeds. Foods.

[27] Ryan E., Galvin K., O’Connor T.P., Maguire A.R., O’Brien N.M. (2007). Phytosterol, Squalene, Tocopherol Content and Fatty Acid Profile of Selected Seeds, Grains, and Legumes. Plant Foods Hum. Nutr..

[28] Rodríguez Madrera R., Campa Negrillo A., Ferreira Fernández J.J. (2024). Modulation of the Nutritional and Functional Values of Common Bean by Farming System: Organic vs. Conventional. Front. Sustain. Food Syst..

[29] Puerta Romero J. (1961). Variedades de Judía Cultivadas en España. Monografía Nº 11.

[30] Pérez-Vega E., Campa A., De la Rosa L., Giraldez R., Ferreira J.J. (2009). Genetic diversity in a core collection established from the main bean genebank in Spain. Crop Sci..

[31] Santalla M., Rodiño A., De Ron A. (2002). Allozyme evidence supporting southwestern Europe as a secondary center of genetic diversity for the common bean. Theor. Appl. Genet..

[32] Campa A., Murube E., Ferreira J.J. (2018). Genetic Diversity, Population Structure, and Linkage Disequilibrium in a Spanish Common Bean Diversity Panel Revealed through Genotyping-by-Sequencing. Genes.

[33] García-Fernández C., Campa A., Garzón A.S., Miklas P., Ferreira J.J. (2021). GWAS of pod morphological and color characters in common bean. BMC Plant Biol..

[34] Kruijer W., Boer M.P., Malosetti M. (2015). Marker-based estimation of heritability in immortal populations. Genetics.

[35] R Core Team (2023). The R Project Statistical Computing. http://www.Rprojectorg/.

[36] Rodrıguez-Ruiz J., Belarbi E.-H. (1998). Rapid Simultaneous Lipid Extraction and Transesterification for Fatty Acid Analyses. Biotechnol. Tech..

[37] Weston T.R., Derner J.D., Murrieta C.M., Rule D.C., Hess B.W. (2008). Comparison of Catalysts for Direct Transesterification of Fatty Acids in Freeze-Dried Forage Samples. Crop Sci..

[38] Miller J.N. (1991). Basic Statistical Methods for Analytical Chemistry Part 2. Calibration and Regression Methods. A Review. Analyst.

[39] FAO, FAO (2004). Food Energy: Methods of Analysis and Conversion Factors; Report of a Technical Workshop, Rome, 3—6 December 2002.

[40] Møller A. (2011). Fatty Acid Molecular Weights and Conversion Factors.

[41] Youden W.J., Steiner E.H. (1975). Statistical Manual of the Association of Official Analytical Chemists.

[42] Calder P.C. (2015). Functional Roles of Fatty Acids and Their Effects on Human Health. J. Parenter. Enter. Nutr..

[43] Kaur N., Chugh V., Gupta A.K. (2014). Essential Fatty Acids as Functional Components of Foods- a Review. J. Food Sci. Technol..

[44] Mariamenatu A.H., Abdu E.M. (2021). Overconsumption of Omega-6 Polyunsaturated Fatty Acids (PUFAs) versus Deficiency of Omega-3 PUFAs in Modern-Day Diets: The Disturbing Factor for Their “Balanced Antagonistic Metabolic Functions” in the Human Body. J. Lipids.

[45] Balouchi H., Soltani Khankahdani V., Moradi A., Gholamhoseini M., Piri R., Heydari S.Z., Dedicova B. (2023). Seed Fatty Acid Changes Germination Response to Temperature and Water Potentials in Six Sesame (*Sesamum indicum* L.) Cultivars: Estimating the Cardinal Temperatures. Agriculture.

[46] Izquierdo N., Benech-Arnold R., Batlla D., Belo R.G., Tognetti J., Ahmad P. (2017). Seed Composition in Oil Crops: Its Impact on Seed Germination Performance. Oilseed Crops.

[47] Grela E.R., Günter K.D. (1995). Fatty Acid Composition and Tocopherol Content of Some Legume Seeds. Anim. Feed Sci. Technol..

[48] Ciurescu G., Toncea I., Rapota M., Habeanu M. (2018). Seeds Composition and their Nutrients Quality of Some Pea (*Pisum sativum* L.) and Lentil (*Lens culinaris* Medik.) Cultivars. Rom. Agric. Res..

[49] Gopala Krishna A.G., Prabhakar J.V., Aitzetmüller K. (1997). Tocopherol and Fatty Acid Composition of Some Indian Pulses. J. Am. Oil Chem. Soc..

[50] Hall C., Hillen C., Garden Robinson J. (2017). Composition, Nutritional Value, and Health Benefits of Pulses. Cereal Chem..

[51] Fahmy H.A., El-Shamy S., Farag M.A. (2023). Comparative GC–MS Based Nutrients Profiling of Less Explored Legume Seeds of Melilotus, Medicago, Trifolium, and Ononis Analysed Using Chemometric Tools. Sci. Rep..

[52] Zia-Ul-Haq M., Ahmad M., Iqbal S., Ahmad S., Ali H. (2007). Characterization and Compositional Studies of Oil from Seeds of Desi Chickpea (*Cicer arietinum* L.) Cultivars Grown in Pakistan. J. Am. Oil Chem. Soc..

[53] Tresina Soris P., Mohan V.R. (2011). Chemical analysis and nutritional assessment of two less known pulses of genus Vigna. Trop. Subtrop. Agroecosyst..

[54] Hernández-López V.M., Vargas-Vázquez M.L.P., Muruaga-Martínez J.S., Hernández-Delgado S. (2013). Origin, domestication and diversification of common beans. Advences and perspectives. Rev. Fitotec. Mex..

[55] Sarzynski T., Bertrand B., Rigal C., Marraccini P., Vaast P., Georget F., Campa C., Abdallah C., Nguyen C.T.Q., Nguyen H.P. (2023). Genetic-environment Interactions and Climatic Variables Effect on Bean Physical Characteristics and Chemical Composition of *Coffea arabica*. J. Sci. Food Agric..

