# Supplementary material for: Fatty Acids in Dry Beans (Phaseolus vulgaris L.): A Contribution to Their Analysis and the Characterization of a Diversity Panel
Source: Foods. 2024 Jun 26;13(13):2023. doi: 10.3390/foods13132023 (PMC11241050; doi:10.3390/foods13132023)
Supplement: Supplementary file 1 [file foods-13-02023-s001.zip › Supplementary Figure 1.doc]

Supplementary Figure 1. Concentration (mg/g) and recovery (%) of exhaustive extraction of palmitic acid (C16:0), stearic acid (C18:0), oleic acid (C18:1 ω-9), cis-vaccenic acid (C18:1 ω-7), linoleic acid (C18:2 ω-6) and linolenic acid (C18:3 ω-3) in dry beans of lines Cornell49242 and A4804.
