# Supplementary material for: Fatty Acids in Dry Beans (Phaseolus vulgaris L.): A Contribution to Their Analysis and the Characterization of a Diversity Panel
Source: Foods. 2024 Jun 26;13(13):2023. doi: 10.3390/foods13132023 (PMC11241050; doi:10.3390/foods13132023)
Supplement: Supplementary file 1 [file foods-13-02023-s001.zip › Supplementary Table 1.doc]

Supplementary Table1. Major fatty acids and total fatty acids at the levels of the factorial design. Data expressed in mg/g and in brackets expressed as %.

| Sample code^a^ | Palmitic acid | Stearic  acid | Oleic  acid | cis-vaccenic acid | Linoleic  acid | Linolenic acid | Total fatty acids |
| --- | --- | --- | --- | --- | --- | --- | --- |
| 50-15-80 | 2.33 (20.7) | 0.27 (2.4) | 1.10 (9.7) | 0.26 (2.3) | 2.39 (21.2) | 4.94 (43.8) | 11.29 (100) |
| 50-15-80 | 2.18 (21.0) | 0.24 (2.4) | 1.02 (9.8) | 0.24 (2.3) | 2.22 (21.4) | 4.49 (43.2) | 10.40 (100) |
| 150-15-80 | 1.95 (21.9) | 0.20 (2.2) | 0.87 (9.7) | 0.20 (2.3) | 1.92 (21.5) | 3.80 (42.4) | 8.94 (100) |
| 150-15-80 | 1.93 (21.8) | 0.20 (2.2) | 0.87 (9.9) | 0.20 (2.3) | 1.88 (21.3) | 3.75 (42.4) | 8.84 (100) |
| 300-15-80 | 1.44 (22.8) | 0.15 (2.3) | 0.62 (9.8) | 0.15 (2.3) | 1.32 (21.0) | 2.63 (41.8) | 6.29 (100) |
| 300-15-80 | 1.55 (23.6) | 0.15 (2.3) | 0.66 (10) | 0.15 (2.3) | 1.38 (21.0) | 2.68 (40.7) | 6.59 (100) |
| 50-30-80 | 2.62 (17.7) | 0.32 (2.1) | 1.40 (9.5) | 0.34 (2.3) | 3.16 (21.3) | 6.96 (47.0) | 14.8 (100) |
| 50-30-80 | 2.60 (17.4) | 0.29 (1.9) | 1.40 (9.4) | 0.35 (2.3) | 3.14 (21.1) | 7.14 (47.9) | 14.92 (100) |
| 150-30-80 | 2.65 (17.7) | 0.29 (1.9) | 1.37 (9.2) | 0.34 (2.3) | 3.22 (21.5) | 7.10 (47.4) | 14.98 (100) |
| 150-30-80 | 2.53 (17.7) | 0.27 (1.9) | 1.36 (9.5) | 0.34 (2.3) | 3.06 (21.3) | 6.77 (47.3) | 14.32 (100) |
| 300-30-80 | 2.36 (18.7) | 0.25 (2.0) | 1.20 (9.5) | 0.30 (2.3) | 2.67 (21.2) | 5.85 (46.3) | 12.63 (100) |
| 300-30-80 | 2.42 (18.8) | 0.25 (2.0) | 1.22 (9.4) | 0.29 (2.3) | 2.74 (21.3) | 5.97 (46.3) | 12.9 (100) |
| 50-60-80 | 2.68 (17.5) | 0.32 (2.1) | 1.37 (8.9) | 0.35 (2.3) | 3.30 (21.5) | 7.34 (47.8) | 15.38 (100) |
| 50-60-80 | 2.63 (17.4) | 0.29 (1.9) | 1.42 (9.4) | 0.36 (2.4) | 3.14 (20.8) | 7.28 (48.2) | 15.11 (100) |
| 150-60-80 | 2.73 (17.3) | 0.30 (1.9) | 1.48 (9.4) | 0.36 (2.3) | 3.35 (21.3) | 7.52 (47.8) | 15.74 (100) |
| 150-60-80 | 2.70 (17.3) | 0.30 (1.9) | 1.50 (9.6) | 0.36 (2.3) | 3.34 (21.4) | 7.40 (47.5) | 15.60 (100) |
| 300-60-80 | 2.62 (17.3) | 0.28 (1.9) | 1.42 (9.4) | 0.35 (2.3) | 3.24 (21.4) | 7.21 (47.7) | 15.12 (100) |
| 300-60-80 | 2.60 (17.6) | 0.28 (1.9) | 1.41 (9.6) | 0.34 (2.3) | 3.10 (21.0) | 7.01 (47.5) | 14.75 (100) |
| 50-120-80 | 2.55 (17.3) | 0.29 (2.0) | 1.41 (9.6) | 0.34 (2.3) | 3.09 (21.0) | 7.04 (47.8) | 14.72 (100) |
| 50-120-80 | 2.72 (17.3) | 0.31 (2.0) | 1.48 (9.4) | 0.37 (2.4) | 3.40 (21.6) | 7.44 (47.3) | 15.72 (100) |
| 150-120-80 | 2.71 (17.6) | 0.29 (1.9) | 1.47 (9.5) | 0.36 (2.3) | 3.33 (21.6) | 7.26 (47.1) | 15.42 (100) |
| 150-120-80 | 2.69 (17.4) | 0.29 (1.9) | 1.49 (9.6) | 0.36 (2.3) | 3.31 (21.4) | 7.36 (47.5) | 15.50 (100) |
| 300-120-80 | 2.57 (17.3) | 0.28 (1.9) | 1.43 (9.7) | 0.35 (2.3) | 3.18 (21.4) | 7.03 (47.4) | 14.83 (100) |
| 300-120-80 | 2.58 (17.3) | 0.28 (1.9) | 1.43 (9.6) | 0.35 (2.3) | 3.20 (21.5) | 7.07 (47.4) | 14.90 (100) |
| 50-15-100 | 2.63 (17.9) | 0.30 (2.1) | 1.42 (9.7) | 0.35 (2.4) | 3.13 (21.3) | 6.86 (46.7) | 14.71 (100) |
| 50-15-100 | 2.71 (17.8) | 0.3 0(2,0) | 1.43 (9.4) | 0.36 (2.4) | 3.25 (21.4) | 7.14 (47.0) | 15.19 (100) |
| 150-15-100 | 2.54 (18.5) | 0.27 (1.9) | 1.33 (9.7) | 0.31 (2.3) | 2.92 (21.3) | 6.37 (46.4) | 13.73 (100) |
| 150-15-100 | 2.62 (18.3) | 0.28 (2,0) | 1.37 (9.6) | 0.34 (2.4) | 2.99 (21.0) | 6.67 (46.7) | 14.27 (100) |
| 300-15-100 | 2.22 (19.9) | 0.23 (2.1) | 1.09 (9.7) | 0.26 (2.3) | 2.39 (21.4) | 4.99 (44.7) | 11.18 (100) |
| 300-15-100 | 2.35 (18.8) | 0.25 (2,0) | 1.18 (9.4) | 0.29 (2.3) | 2.66 (21.3) | 5.78 (46.2) | 12.52 (100) |
| 50-30-100 | 2.68 (17.4) | 0.30 (2,0) | 1.50 (9.8) | 0.36 (2.3) | 3.23 (21.0) | 7.30 (47.5) | 15.38 (100) |
| 50-30-100 | 2.72 (17.5) | 0.32 (2,0) | 1.42 (9.1) | 0.37 (2.4) | 3.36 (21.6) | 7.36 (47.4) | 15.54 (100) |
| 150-30-100 | 2.64 (17.5) | 0.29 (1.9) | 1.44 (9.5) | 0.35 (2.3) | 3.20 (21.2) | 7.19 (47.6) | 15.11 (100) |
| 150-30-100 | 2.76 (17.6) | 0.30 (1.9) | 1.46 (9.3) | 0.36 (2.3) | 3.36 (21.4) | 7.49 (47.6) | 15.72 (100) |
| 300-30-100 | 2.61 (18.0) | 0.29 (2.0) | 1.40 (9.7) | 0.34 (2.3) | 3.09 (21.3) | 6.79 (46.8) | 14.52 (100) |
| 300-30-100 | 2.64 (17.9) | 0.29 (1.9) | 1.42 (9.6) | 0.34 (2.3) | 3.15 (21.4) | 6.91 (46.9) | 14.74 (100) |
| 50-60-100 | 2.67 (17.4) | 0.31 (2.0) | 1.44 (9.4) | 0.35 (2.3) | 3.30 (21.5) | 7.28 (47.4) | 15.36 (100) |
| 50-60-100 | 2.68 (17.5) | 0.31 (2.0) | 1.48 (9.7) | 0.36 (2.4) | 3.21 (20.9) | 7.28 (47.5) | 15.32 (100) |
| 150-60-100 | 2.84 (17.6) | 0.31 (1.9) | 1.50 (9.3) | 0.37 (2.3) | 3.45 (21.4) | 7.67 (47.5) | 16.14 (100) |
| 150-60-100 | 2.70 (17.2) | 0.30 (1.9) | 1.46 (9.3) | 0.35 (2.3) | 3.45 (22.0) | 7.43 (47.3) | 15.69 (100) |
| 300-60-100 | 2.71 (17.6) | 0.30 (1.9) | 1.48 (9.6) | 0.36 (2.3) | 3.28 (21.3) | 7.27 (47.2) | 15.39 (100) |
| 300-60-100 | 2.71 (17.5) | 0.30 (1.9) | 1.46 (9.4) | 0.36 (2.3) | 3.30 (21.3) | 7.38 (47.6) | 15.50 (100) |
| 50-120-100 | 2.71 (17.4) | 0.31 (2.0) | 1.48 (9.5) | 0.36 (2.3) | 3.29 (21.2) | 7.38 (47.5) | 15.53 (100) |
| 50-120-100 | 2.59 (17.5) | 0.30 (2.0) | 1.40 (9.5) | 0.35 (2.4) | 3.10 (20.9) | 7.06 (47.7) | 14.81 (100) |
| 150-120-100 | 2.78 (17.4) | 0.31 (1.9) | 1.51 (9.4) | 0.37 (2.3) | 3.40 (21.2) | 7.64 (47.7) | 16.01 (100) |
| 150-120-100 | 2.78 (17.3) | 0.30 (1.9) | 1.53 (9.5) | 0.37 (2.3) | 3.45 (21.5) | 7.64 (47.5) | 16.07 (100) |
| 300-120-100 | 2.65 (17.5) | 0.29 (1.9) | 1.47 (9.8) | 0.35 (2.4) | 3.20 (21.2) | 7.12 (47.2) | 15.09 (100) |
| 300-120-100 | 2.71 (17.6) | 0.29 (1.9) | 1.45 (9.5) | 0.36 (2.3) | 3.28 (21.3) | 7.28 (47.4) | 15.38 (100) |
| Factor |  |  |  |  |  |  |  |
| M | * | * | * | * | * | * | * |
| tt | * | * | * | * | * | * | * |
| T | * | * | * | * | * | * | * |
| M x tt | * | * | * | * | * | * | * |
| M x T | * | * | * | * | * | * | * |
| tt x T | * | * | * | * | * | * | * |
| M x tt x T | n.s. | n.s. | n.s. | n.s. | n.s. | n.s. | n.s. |

^a^Sample code: mg_min_ºC; M: sample mass; tt: transesterification time; T: temperature; *: significant differences (p<0.01); n.s.: not significant
