# Supplementary material for: Fatty Acids in Dry Beans (Phaseolus vulgaris L.): A Contribution to Their Analysis and the Characterization of a Diversity Panel
Source: Foods. 2024 Jun 26;13(13):2023. doi: 10.3390/foods13132023 (PMC11241050; doi:10.3390/foods13132023)
Supplement: Supplementary file 1 [file foods-13-02023-s001.zip › Supplementary Table 2.doc]

Supplementary Tale 2. Analytical characteristics of the calibration graphs of major fatty acids in dry beans.

|  | Palmitic acid (μg/mL) | Stearic acid (μg/mL) | Oleic acid (μg/mL) | cis-Vaccenic acid (μg/mL) | Nonadecanoic acid* (μg/mL) | Linoleic acid (μg/mL) | Linolenic acid (μg/mL) |
| --- | --- | --- | --- | --- | --- | --- | --- |
| **Range** | 2-612 | 2-200 | 2-650 | 2-200 | 2-200 | 2-1,274 | 2-1,216 |
| **n** | 9 | 7 | 9 | 7 | 7 | 9 | 9 |
| **m** | 48,632.5 | 49,054.3 | 47,938.8 | 48,889.1 | 49,193.7 | 48,982.7 | 47,928.1 |
| **a** | 128,511.3 | 45,037.4 | -374.0 | -3,953.8 | 62,910.4 | -47,062.6 | -35,203.5 |
| **R^2^** | 0.9998 | 0.9999 | 0.9991 | 0.9992 | 1.0000 | 1.0000 | 0.9995 |

*: Internal standard. n: number of points in calibration curve. m: slope, a: intercept; R^2^: correlation coefficient.
