# Supplementary material for: Fatty Acids in Dry Beans (Phaseolus vulgaris L.): A Contribution to Their Analysis and the Characterization of a Diversity Panel
Source: Foods. 2024 Jun 26;13(13):2023. doi: 10.3390/foods13132023 (PMC11241050; doi:10.3390/foods13132023)
Supplement: Supplementary file 1 [file foods-13-02023-s001.zip › Supplementary Table 3.doc]

Supplementary Table 3. Repeatability and reproducibility of the method under the optimized conditions.

|  | Palmitic  acid | Stearic  acid | Oleic  acid | cis-Vaccenic acid | Linoleic  acid | Linolenic  acid | Total fatty acids |
| --- | --- | --- | --- | --- | --- | --- | --- |
| *Operator 1* |  |  |  |  |  |  |  |
| Mean value (mg/g, n=3) | 2.78 | 0.31 | 1.52 | 0.37 | 3.38 | 7.52 | 15.88 |
| Repeatability, r (RSD, %) | 0.35 | 3.20 | 0.88 | 0.53 | 0.93 | 1.02 | 0.69 |
| *Operator 2* |  |  |  |  |  |  |  |
| Mean value (mg/g, n=3) | 2.62 | 0.28 | 1.44 | 0.35 | 3.20 | 7.12 | 15.01 |
| Repeatability, r (RSD, %) | 1.44 | 1.27 | 1.17 | 1.95 | 0.59 | 0.91 | 0.97 |
|  |  |  |  |  |  |  |  |
| Reproducibility, R (mg/g, RSD, %) | 4.13 | 5.57 | 4.08 | 4.72 | 3.94 | 3.86 | 3.75 |

RSD: Relative standard deviation.
