# Supplementary material for: Fatty Acids in Dry Beans (Phaseolus vulgaris L.): A Contribution to Their Analysis and the Characterization of a Diversity Panel
Source: Foods. 2024 Jun 26;13(13):2023. doi: 10.3390/foods13132023 (PMC11241050; doi:10.3390/foods13132023)
Supplement: Supplementary file 1 [file foods-13-02023-s001.zip › Supplementary Table 4.doc]

Supplementary Table 4. Method accuracy in samples from interlaboratory assays.

|  | Reference value (sigma) | TFA as TAG (%)* | \|Z-Score\| | Accuracy (%) |
| --- | --- | --- | --- | --- |
| Corn silage | 2.66 (0.45) | 2.44 | 0.49 | 92 |
| Bran | 4.36 (0.28) | 4.77 | 1.46 | 109 |
| DDG** | 7.52 (0.49) | 8.20 | 1.39 | 109 |
| Palm kernel | 6.00 (0.48) | 6.59 | 1.23 | 110 |
| Grass sillage | 2.76 (0.22) | 2.75 | 0.05 | 100 |
| Sorghum | 2.97 (0.26) | 3.34 | 1.42 | 112 |

TFA: total fatty acids; TAG: Triacylglicerydes; *: calculated according to Møller [40]; **: Dried distillers grain.
