# Supplementary material for: Fatty Acids in Dry Beans (Phaseolus vulgaris L.): A Contribution to Their Analysis and the Characterization of a Diversity Panel
Source: Foods. 2024 Jun 26;13(13):2023. doi: 10.3390/foods13132023 (PMC11241050; doi:10.3390/foods13132023)
Supplement: Supplementary file 1 [file foods-13-02023-s001.zip › Supplementary Table 5.doc]

Supplementary Table 5. Youden-Steiner test scheme and results of the analysis of the major fatty acids under these conditions.

|  | Determination number | | | | | | | |
| --- | --- | --- | --- | --- | --- | --- | --- | --- |
| Factor Value | 1 | 2 | 3 | 4 | 5 | 6 | 7 | 8 |
| A-Sample mass (mg; A=150. a=200) | A | A | A | A | a | a | a | a |
| B-Heating system (B=Bath water. b=Oven) | B | B | b | b | B | B | b | b |
| C-HCl/Methanol stability (C=Fresh. c= 1 month) | C | c | C | c | C | c | C | c |
| D-Volume hexane (mL; D=1. d=2) | D | D | d | d | d | d | D | D |
| E-Volume HCl/Methanol (mL; E=2. e=3) | E | e | E | e | e | E | e | E |
| F-Internal standard (µg; F=200. f=400) | F | f | f | F | F | f | f | F |
| G-Chromatographic equipment (G=Agilent 7890 column 1. Agilent 6890 column 2) | G | g | g | G | g | G | G | g |
| *Observed result* | *s* | *t* | *u* | *v* | *w* | *x* | *y* | *z* |
| Palmitic acid | 3.75 | 3.67 | 3.72 | 3.69 | 3.70 | 3.41 | 3.73 | 3.6 |
| Stearic acid | 0.48 | 0.43 | 0.44 | 0.46 | 0.43 | 0.40 | 0.46 | 0.42 |
| Oleic acid | 2.02 | 2.00 | 2.04 | 2.02 | 2.02 | 1.77 | 2.08 | 1.96 |
| cis-Vaccenic acid | 0.32 | 0.31 | 0.31 | 0.32 | 0.31 | 0.27 | 0.33 | 0.31 |
| Linoleic acid | 7.82 | 7.78 | 7.86 | 7.75 | 7.74 | 6.44 | 8.05 | 7.50 |
| Linolenic acid | 5.84 | 5.73 | 5.86 | 5.74 | 5.75 | 4.72 | 5.98 | 5.63 |
| Total fatty acids | 20.83 | 20.5 | 20.83 | 20.58 | 20.46 | 17.56 | 21.24 | 19.99 |
